# Supplementary material for: Differentiating Real-World Autobiographical Experiences without Recourse to Behaviour
Source: Brain Sci. 2021 Apr 20;11(4):521. doi: 10.3390/brainsci11040521 (PMC8074167; doi:10.3390/brainsci11040521)
Supplement: Supplementary file 1 [file brainsci-11-00521-s001.zip › brainsci-1144371-supplementary.pdf]

Supplementary Material

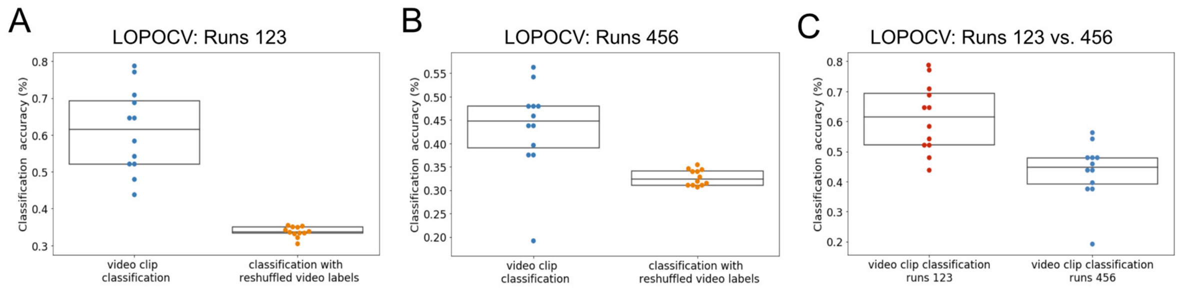

Supplementary Figure S1. LOPOCV Analyses.

a) *Classification using the first 3 runs.* The mean classification accuracy using only the first 3 runs was 0.61 (SD = 0.11) (Supplementary Figure 1A). The same analysis with reshuffled video labels yielded a mean classification accuracy of 0.34 (SD = 0.01). A paired t-test between the two distributions indicated that they were significantly different from one another ($t = 8.55$, $p < 0.001$).

b) *Classification using the last 3 runs.* The mean classification accuracy using only the last 3 runs was 0.43 (SD = 0.10) (Supplementary Figure 1B). The same analysis with reshuffled video labels yielded a mean classification accuracy of 0.33 (SD = 0.02). A paired t-test between the two distributions indicated that they were significantly different from one another ($t = 3.93$, $p < 0.005$).

c) *Comparing classification accuracyoif the first 3 runs vs. last 3 runs.* The classification accuracy using only the data from the first 3 runs was significantly higher than that using the last 3 runs ($t = 3.46$, $p < 0.01$; Supplementary Figure 1C). This is not surprising, as by the last 3 runs all videos were familiar (were presented once already) which should make differentiating between them harder.

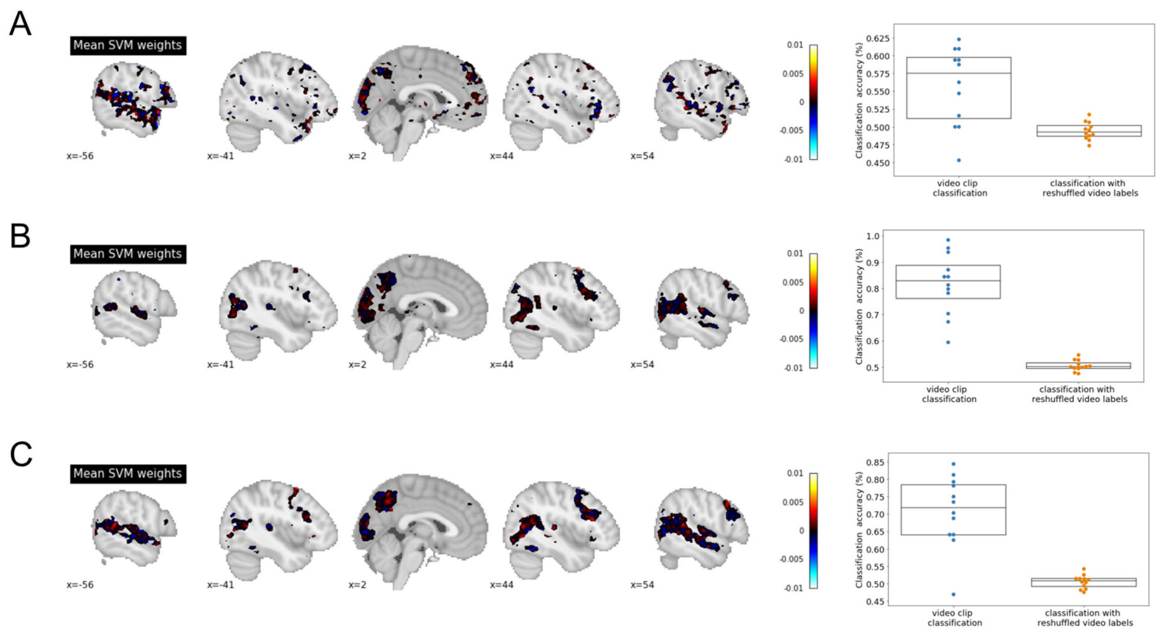

Supplementary Figure S2. 2-way classifications.
In addition to the 3-way classification analyses, we also ran additional analyses looking at classification performance when only 2 categories were included. All 3 two-way classification analyses (*Own vs. Other*, *Own vs. Bookstore* and *Other vs. Bookstore*) yielded classification accuracies that were higher than chance.
(A) *Own vs. Other.* The mean classifier accuracy across participants was 0.56 (SD = 0.05). In order to determine whether this was above chance, the same analysis was repeated with randomly reshuffled video labels. The classification accuracies with the reshuffled labels are depicted in orange. The mean classifier accuracy across participants for these reshuffled labels was 0.49 (SD = 0.01). A paired t-test between the two distributions (blue dots vs. orange dots) indicated that they were significantly different from each other ($t = 3.64$, $p < 0.005$).
(B) *Own vs. Bookstore*. Classification accuracy was 0.82 (SD = 0.12), and higher than classification accuracy with reshuffled labels (M = 0.5, SD = 0.02; $t = 8.55$, $p < 0.001$). This is expected as the events differ in both the mnemonic status and the visual appearance, as well as semantic content.
(C) The *Other vs. Bookstore* classification differs in terms of the visual content, but is similar in terms of the mnemonic status of the events. Mean classification was 0.71, SD = 0.10. Classification with reshuffled labels was 0.51 (SD = 0.02). This difference is significant ($t = 7.34$, $p < 0.001$).

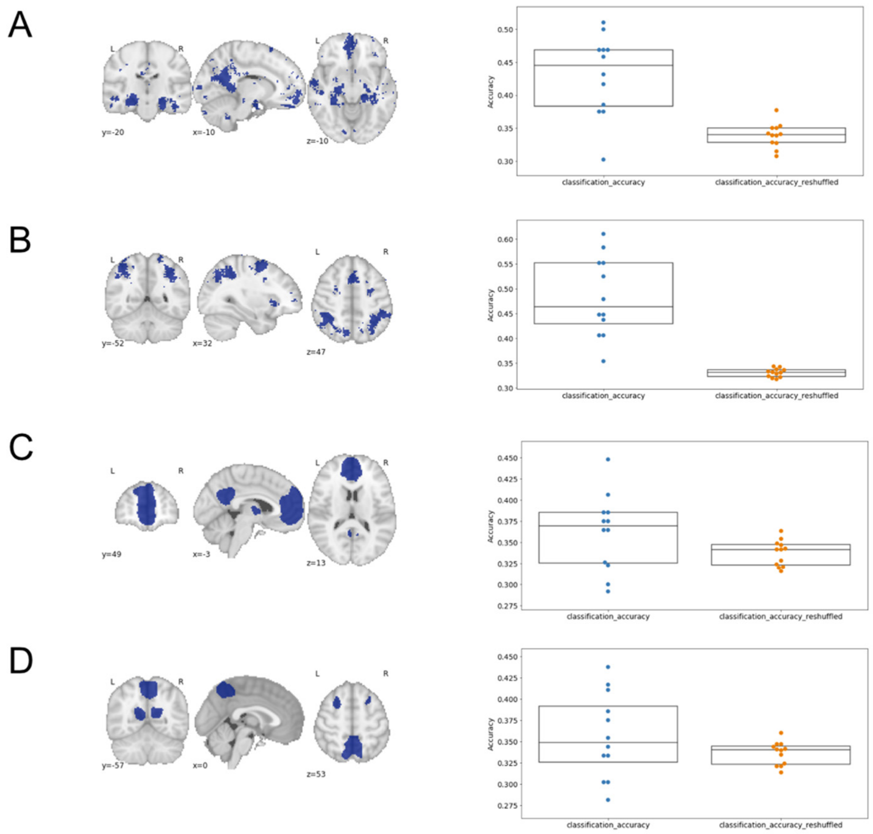

Supplementary Figure S3. Classifications using masks.

In addition to the analysis conducted with the Autobiographical mask, we also conducted 3 additional analyses using a fronto-parietal mask, as well as ventral DMN and dorsal DMN masks. The fronto-parietal mask was defined via Neurosynth, a platform for large-scale, meta-analysis of fMRI data from published studies (http://neurosynth.org/; (Yarkoni et al., 2011). The search term we used was “frontoparietal”, which yielded 360 studies that included 13,467 activations. The dorsal and ventral DMN masks were defined by templates provided by Stanford's Functional Imaging in Neuropsychiatric Disorders lab (Shirer et al., 2012). These analyses revealed that classification accuracy was above chance when using voxels restricted to **(A)** the autobiographical memory mask ($M = 0.43$ ($SD = 0.06$), $M_{reshuffled} = 0.34$ ($SD = 0.02$); $t = 4.90$, $p < 0.001$) and **(B)** the fronto-parietal mask ($M = 0.48$ ($SD = 0.08$), $M_{reshuffled} = 0.33$ ($SD = 0.01$), $t = 6.27$, $p < 0.001$.), but not **(C)** the dorsal DMN mask (0.36 ($SD = 0.05$), 0.34, ($SD = 0.02$), $t = 1.70$, $p > 0.05$) or **(D)** the ventral DMN mask ($M = 0.36$ ($SD = 0.05$), $M_{reshuffled} = 0.34$ ($SD = 0.01$), $t = 1.50$, $p > 0.05$).

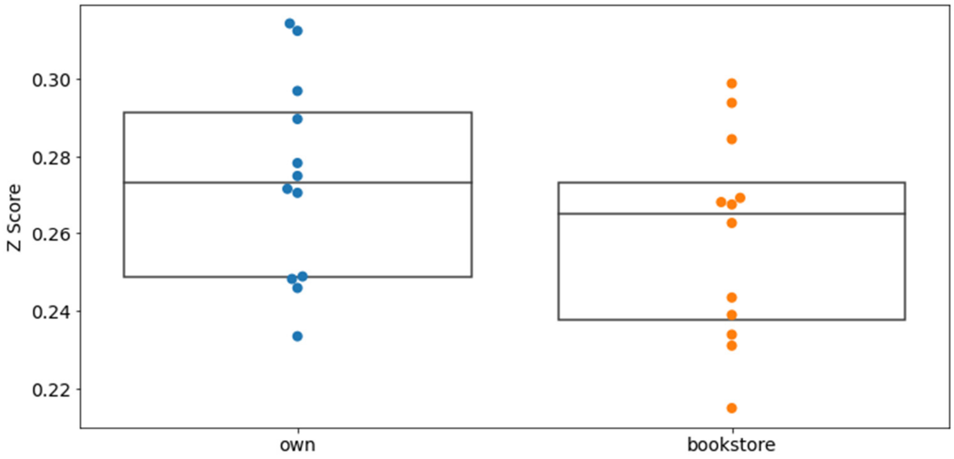

Supplementary Figure S4. Z-scored model feature weights using autobiographical memory mask. Here, model feature-weights were converted to z-scores for each participant. The voxel-wise z-scores were then masked using the Neurosynth Autobiographical Memory mask, such that each dot represents the mean z -score across all voxels in the mask. The average z-scores were then evaluated for statistics significance with a paired-samples t -test. This analysis revealed that z scores for the *Own* condition were significantly higher than the *Bookstore* condition ($M_{Own} =$ 0.274 (SD = 0.03), $M_{Bookstore} =$ 0.259 (SD = 0.03); $t = 4.23$, $p < 0.005$).

WPCV Analyses

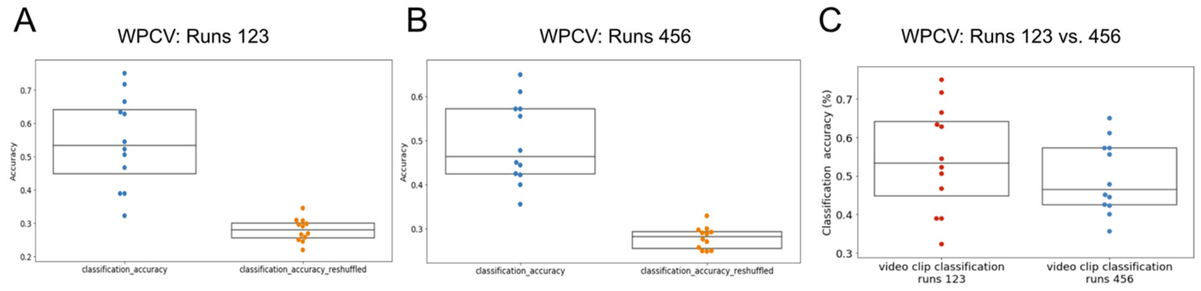

Supplementary Figure S5. WPCV Analyses.

a) *Classification using the first 3 runs.* The mean classification accuracy using only the first 3 runs was 0.54 (SD = 0.14) (Supplementary Figure 4A). The same analysis with reshuffled video labels yielded a mean classification accuracy of 0.28 (SD = 0.03). A paired t-test between the two distributions indicated that they were significantly different from one another ($t = 6.36$, $p < 0.001$).

b) *Classification using the last 3 runs.* The mean classification accuracy using only the last 3 runs was 0.49 (SD = 0.09) (Supplementary Figure 4B). The same analysis with reshuffled video labels yielded a mean classification accuracy of 0.28 (SD = 0.03). A paired t-test between the two distributions indicated that they were significantly different from one another ($t = 7.22$, $p < 0.001$).

c) *Comparing classification accuracy of the first 3 runs vs. last 3 runs.* The classification accuracy using only the data from the first 3 runs was not different than the classification accuracy when using only the last 3 runs ($t = 1.23$, $p = 0.25$).

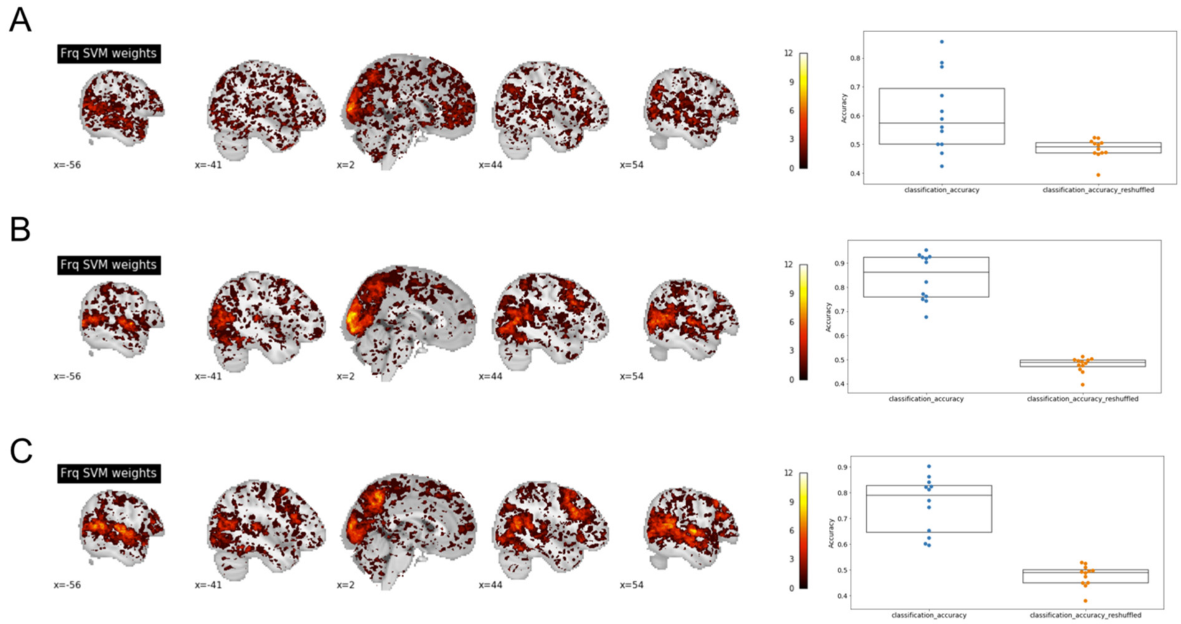

Supplementary Figure S6. 2-way classifications.
In addition to the 3-way classification analyses, we also ran additional analyses looking at classification performance when only 2 categories were included. All 3 two-way classification analyses (*Own vs. Other*, *Own vs. Bookstore* and *Other vs. Bookstore*) yielded classification accuracies that were higher than chance.
(A) *Own vs. Other.* The mean classifier accuracy across participants was 0.61 (SD = 0.14). In order to determine whether this was above chance, the same analysis was repeated with randomly reshuffled video labels. The classification accuracies with the reshuffled labels are depicted in orange. The mean classifier accuracy across participants for these reshuffled labels was 0.48, (SD = 0.04). A paired t-test between the two distributions (blue dots vs. orange dots) indicated that they were significantly different from each other ($t = 2.99$, $p < 0.05$).
(B) *Own vs. Bookstore.* Classification accuracy was 0.84 (SD = 0.10), and higher than classification accuracy with reshuffled labels (M = 0.48, SD = 0.03; $t = 12.31$, $p < 0.001$).
(C) The *Other vs. Bookstore* classification differs in terms of the visual content, but is similar in terms of the mnemonic status of the events. Mean classification was 0.75, SD = 0.11. Classification with reshuffled labels was 0.48 (SD = 0.04). This difference is significant ($t = 10.08$, $p < 0.001$).

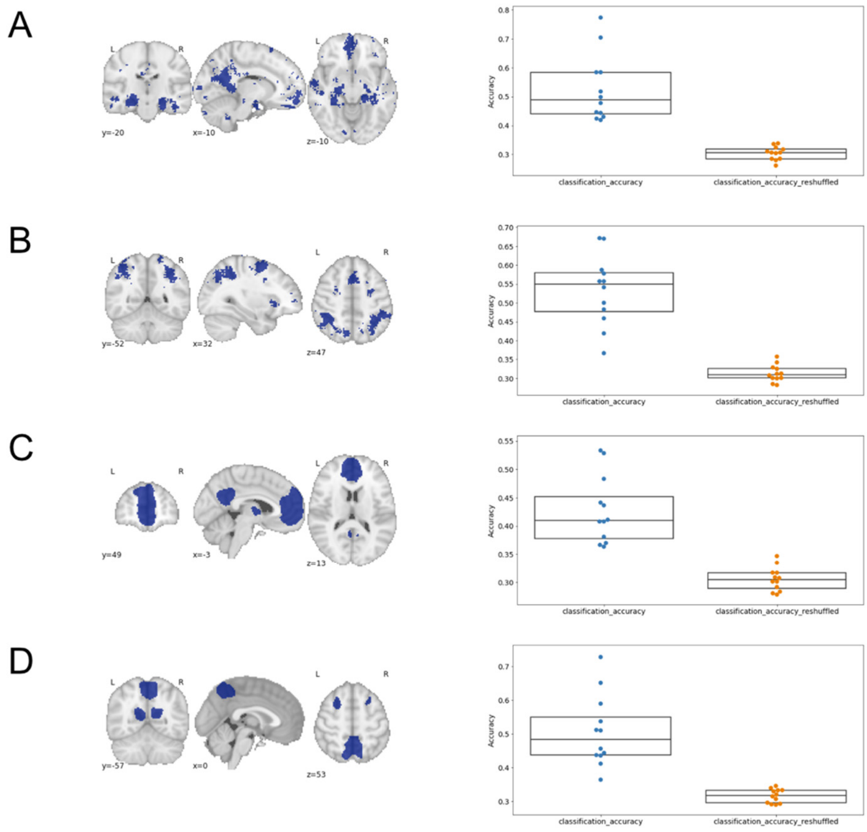

Supplementary Figure S7. Classifications using pre-defined masks.
These analyses revealed that classification accuracy was above chance when using voxels restricted to all four masks: **(A)** Autobiographical memory mask (M = 0.53 (SD = 0.12), $M_{reshuffled}$ = 0.30 (SD = 0.02); t = 7.15, p < 0.001), **(B)** the fronto-parietal mask (M = 0.53 (SD = 0.09), $M_{reshuffled}$ = 0.31 (SD = 0.02), t = 9.63, p < 0.001), **(C)** the dorsal DMN mask (0.51 (SD = 0.11), 0.32, (SD = 0.02), t = 6.48, p < 0.001), and **(D)** the ventral DMN mask (M = 0.43 (SD = 0.06), $M_{reshuffled}$ = 0.31 (SD = 0.02), t = 7.51, p < 0.001).

References

Shirer, W. R., Ryali, S., Rykhlevskaia, E., Menon, V., & Greicius, M. D. (2012). Decoding subject-driven cognitive states with whole-brain connectivity patterns. Cerebral cortex, 22(1), 158-165.
